# The putative signal peptide of glucagon-like peptide-1 receptor is not required for receptor synthesis but promotes receptor expression

**DOI:** 10.1042/BSR20140120

**Published:** 2014-11-21

**Authors:** Yunjun Ge, Dehua Yang, Antao Dai, Caihong Zhou, Yue Zhu, Ming-Wei Wang

**Affiliations:** *The National Center for Drug Screening and the CAS Key Laboratory of Receptor Research, Shanghai Institute of Materia Medica, Chinese Academy of Sciences, Shanghai 201203, China; †School of Bioscience and Bioengineering, South China University of Technology, Guangzhou 510641, China

**Keywords:** epitope tag, G protein-coupled receptor, glucagon-like peptide-1 receptor, glycosylation, signal peptide, synthesis, CRF_1_R, corticotropin-releasing factor 1 receptor, CRF_2(a)_R, corticotropin-releasing factor 2a receptor, Endo H, endoglycosidase H, ER, endoplasmic reticulum, FLAG, synthetic epitope tag, GAPDH, glycerinaldehyd-3-phosphatdehydrogenase, GFP, green fluorescent protein, GLP-1, glucagon-like peptide-1, GPCR, G protein-coupled receptor, HA, epitope tag from human influenza haemagglutinin protein, HBSS, Hanks balanced salt solution, HEK293 cell, human embryonic kidney 293 cell, PNGase F, peptide-*N*-glycosidase F, SP, signal peptide

## Abstract

GLP-1R (glucagon-like peptide-1 receptor) mediates the ‘incretin effect’ and many other anti-diabetic actions of its cognate ligand, GLP-1 (glucagon-like peptide-1). It belongs to the class B family of GPCRs (G protein-coupled receptors) and possesses an N-terminal putative SP (signal peptide). It has been reported that this sequence is required for the synthesis of GLP-1R and is cleaved after receptor synthesis. In the present study, we conducted an in-depth exploration towards the role of the putative SP in GLP-1R synthesis. A mutant GLP-1R without this sequence was expressed in HEK293 cells (human embryonic kidney 293 cells) and displayed normal functionality with respect to ligand binding and activation of adenylate cyclase. Thus the putative SP does not seem to be required for receptor synthesis. Immunoblotting analysis shows that the amount of GLP-1R synthesized in HEK293 cells is low when the putative SP is absent. This indicates that the role of the sequence is to promote the expression of GLP-1R. Furthermore, epitopes tagged at the N-terminal of GLP-1R are detectable by immunofluorescence and immunoblotting in our experiments. In conclusion, the present study points to different roles of SP in GLP-1R expression which broadens our understanding of the functionality of this putative SP of GLP-1R and possibly other Class B GPCRs.

## INTRODUCTION

GLP-1R (glucagon-like peptide-1 receptor) is a validated therapeutic target for the treatment of type 2 diabetes. It mediates the ‘incretin effect’ of GLP-1 (glucagon-like peptide-1) and is thus associated with the enhanced glucose-dependent insulin release following glucose intake [1]. It is also involved in other anti-diabetic effects exerted by GLP-1, such as increasing pancreatic β-cell mass, inhibiting glucagon secretion, suppressing appetite and slowing gastric empting, etc. [2,3].

GLP-1R belongs to the class B family of GPCRs (G protein-coupled receptors). It is reported that members of this family probably contain an N-terminal cleavable SP (signal peptide) [4,5]. The latter is a sequence of approximately 20 amino acids in the N-terminal of precursor secreting or membrane proteins [6]. After synthesis of the receptor's N-tail in cytoplasm, an SP stops the translation (‘elongation arrest’) by binding to the SRP (signal recognition particle) [7,8]. The resulting complex is targeted to the ER (endoplasmic reticulum) membrane and the nascent chain transferred to the translocon complex, followed by translocon gating, protein synthesis and nascent chain being integrated into the bilayer [9]. Usually, the SP is cleaved off by a signal peptidase after mediating the ER targeting/insertion process [10]. For most GPCRs, full-length receptors are then glycosylated within extracellular domains during the transportation through the ER and Golgi, where high-mannose oligosaccharides and complex oligosaccharides are added [11,12].

It is known that a majority of GPCRs do not possess an N-terminal SP and the first transmembrane helix (TM1; signal anchor sequence) takes the role of SP [5,11,13]. However, it is not completely understood why some GPCRs require an additional SP but others do not. Such an SP is reported to be essential for the large N-terminal domain of some GPCRs to translocate across the ER membrane after translation [4]. Nonetheless, the putative SPs of rat CRF_1_R (corticotropin-releasing factor 1 receptor) and rat CRF_2(a)_R (corticotropin-releasing factor 2a receptor) are not essential for establishing a functional receptor at the cell surface and the rat CRF_2(a)_R contains an uncleaved N-terminal SP [14,15]. The complexity of these signal sequences highlights the need for experimental verification of the roles that these putative SPs play in other GPCRs.

In case of GLP-1R, there has been one report claiming the presence of an N-terminal cleavable SP and its absolute requirement for receptor synthesis [16]. However, we are able to detect the expression of mutant receptors without the putative SP and find that N-terminal epitope-tags of the GLP-1R are not completely undetectable. Therefore, we conducted an in-depth study on the putative SP of GLP-1R using confocal laser scanning microscopy and immunoblotting analysis in order to identify its roles in the expression of GLP-1R.

## MATERIALS AND METHODS

### Materials

The original plasmids containing cDNA sequence of CRF_1_R or CRF_2(a)_R are the gifts from Dr. Ralf Schülein (Leibniz-Institut für Molekulare Pharmakolgie, Germany). The radiolabelled peptide ^125^I-GLP-1_7-36_ was obtained from PerkinElmer (Boston, MA, U.S.A.). Unlabelled GLP-1_7-36_ amide and RIPA buffer were purchased from Sigma (St. Loius, MO, U.S.A.). Mouse anti-HA (epitope tag from human influenza haemagglutinin protein) antibody, mouse anti-β-actin antibody, rabbit polyclonal anti-GFP (green fluorescent protein) antibody, goat anti-mouse IgG HRP-linked antibody and goat anti-rabbit IgG HRP-linked antibody were provided by Cell Signaling Technology (Danvers, MA, U.S.A.). Mouse anti-FLAG (synthetic epitope tag) antibody was procured from Wako Pure Chemical Industries, Ltd (Osaka, Japan). PageRuler prestained protein ladder was from ThermoFisher Scientific (Rockford, IL, U.S.A.). PNGase F (peptide-*N*-glycosidase F) and Endo H (endoglycosidase H) were bought from New England Biolabs (Ipswich, MA, U.S.A.). SuperSignal® West Dura substrate was purchased from Pierce Biotechnology (Pittsburgh, PA, U.S.A.). Trizol® reagent, Lipofectamine™ 2000, vector pcDNA3.1(+) and oligonucleotides were the products of Invitrogen (Carlsbad, CA, U.S.A.). Vector pd2EGFP-N1 was the product of Clontech Laboratories (Mountain, View, CA, U.S.A.). Alexa Fluor® 488 donkey anti-mouse antibody and CellMask™ Deep Red plasma membrane stain was from Life Technologies (Carlsbad, CA, U.S.A.). Cell dissociation buffer (enzyme-free Hanks’-based) was purchased from Gibco (Grand Island, NY U.S.A.). The cAMP Dynamic 2 Kit was the product of Cisbio International (Codolet France). High Capacity Reverse Transcription Kit was provided by Applied Biosystems (Foster City, CA, U.S.A.). Muta-directed™ Site-Directed Mutagenesis Kit was the product of SBS Genetech Co., Ltd (Beijing, China).

### Generation of cDNA constructs

**Figure 1:**
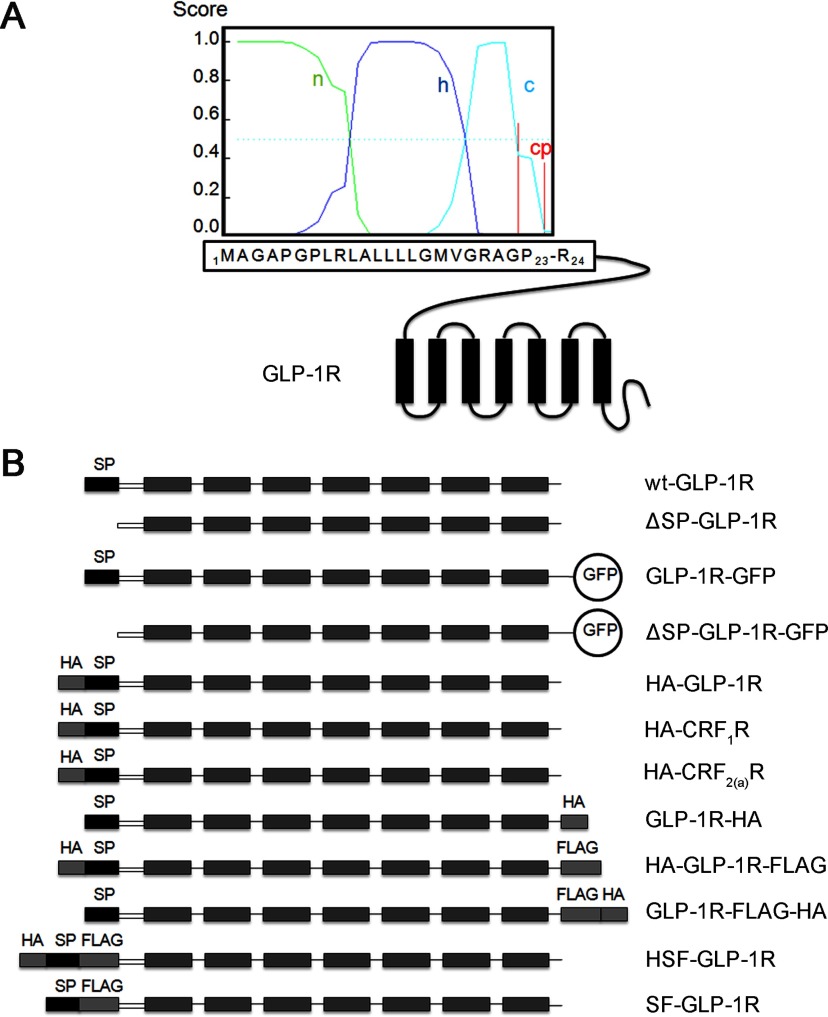
Signal peptide prediction and cDNA construct illustration (**A**) Depiction of the putative signal sequence and the cleavage site. N-terminal amino acids of GLP-1R were monitored with the program ‘SignalP 3.0’. The probabilities of the presence of n (green), h (blue) and c (light blue) regions, and the cleavage probabilities (cp, red) are indicated in a score ranging from 0 to 1. (**B**) Presentation of the constructs used in the present study. The SPs and the transmembrane domains are shown as gray or black boxes. N-tail sequences are indicated as open boxes. HA and FLAG tags are shown as grey boxes. Fused GFP is indicated. Signal peptides of GLP-1R, CRF_1_R and CRF_2(a)_R are the first 23, 24 and 19 amino acids, respectively.

All of the constructs used in this study are shown in Figure 1(B). To generate the construct GLP-1R-GFP, the coding sequence of hGLP-1R (human GLP-1R) with appropriate restriction sites was cloned by PCR and ligated into pd2EGFP-N1. The cDNA of wild-type hGLP-1R in pcDNA3.1(+) was used as a template. For the construct ΔSP-GLP-1R-GFP, forward primer with a start codon was designed to clone the coding sequence of mutant GLP-1R without the first 23 amino acids. The construct ΔSP-GLP-1R was generated by ligating the coding sequence of the mutant GLP-1R into pcDNA3.1(+). The coding sequences of HA/FLAG-tagged hGLP-1R were cloned by PCR with primers containing the nucleotides of HA/FLAG epitope and ligated into pcDNA3.1(+) to generate HA-GLP-1R, GLP-1R-HA and HA-GLP-1R-FLAG. GLP-1R-FLAG was first generated and then used as a template to prepare the construct GLP-1R-FLAG-HA. The constructs, HA-CRF_1_R and HA-CRF_2(a)_R, were generated by the same method. The construct, SF-GLP-1R, was generated by overlap-extension PCR and then used as a template to generate HSF-GLP-1R. P23A-HA-GLP-1R, P23S-HA-GLP-1R, P23A-GLP-1R-GFP and P23S-GLP-1R-GFP were generated using the Muta-directed™ Site-Directed Mutagenesis Kit. All of the plasmids were purified with QIAGEN plasmid kit (Hilden, Germany) and sequences confirmed with DNA sequencing thereafter. Details of the cloning strategies are available upon request.

### Cell culture and transfection

HEK293 cells (human embryonic kidney 293 cells) were cultured in DMEM (Dulbecco's modified Eagle's medium) with 10% (v/v) FBS, 100 units/ml of penicillin and 100 μg/ml streptomycin at 37°C in a 5% (v/v) CO_2_ humidified environment. Cells were cultured at about 70% confluency and transfected using Lipofectamine™ 2000 according to the manufacturer's instructions.

### Receptor binding assay

Whole-cell binding experiments were conducted as described previously [17]. Briefly, cells in 96-well plates were harvested 24 h after transfection, washed three times and incubated with blocking buffer [F12 supplemented with 33 mM HEPES, pH 7.4 and 0.1% (v/v) BSA] for 2 h at 37°C. For homogeneous binding assay, cells were incubated in binding buffer with 200 pM of ^125^I-GLP-1 and different concentrations of unlabelled GLP-1 (21.4 pM–166.7 nM) at room temperature for 3 h. Cells were washed three times and lysed by 50 μl lysis buffer [PBS supplemented with 20 mM Tris–HCl and 1% (v/v) Triton X-100, pH 7.4]. The plates were centrifuged and subsequently counted for radioactivity (counts per minute, CPM) in a scintillation counter (MicroBeta^2^ Plate Counter, PerkinElmer) using a scintillation cocktail (OptiPhase SuperMix, PerkinElmer).

### cAMP measurement

Twenty-four hours after transfection, cell monolayers in 12-well tissue culture plates were washed and treated with cell dissociation buffer. Cells were collected by centrifugation at 200 ***g*** for 3 min and resuspended with HBSS buffer [Hanks balanced salt solution supplemented with 0.1% (w/v) BSA and 5 mM HEPES]. Cells were diluted to a density of 5×10^5^/ml and transferred (10 μl) to the wells of 384-well plates. GLP-1_7–36_ amide in the HBSS buffer (10 μl) at the required concentrations was then added, followed by incubation at room temperature for 30 min. Cells were lysed and cAMP levels were measured by a radioimmunoassay with cAMP Dynamic 2 Kit according to the manufacturer's instructions. The final reaction volume of each well was 40 μl. Time-resolved fluorescence was read at 665 and 615 nm on an EnVision plate reader (PerkinElmer). Values were converted to concentrations of cAMP using a cAMP standard curve performed in parallel.

### Immunoblotting

Twenty-four hours after transfection, HEK293 cells (2.5×10^6^) were lysed with RIPA buffer [50 mM Tris–HCl, pH 8.0, with 150 mM sodium chloride, 1.0% (v/v) NP-40, 0.5% (w/v) sodium deoxycholate, and 0.1% (w/v) sodium dodecyl sulphate]. Each of the cell lysates was divided into five aliquots and one sample was incubated with denaturing buffer at 37°C for 10 min. Protein samples were separated by SDS–PAGE (8% gel) and transferred onto PVDF membranes. After a 2 h incubation with blocking buffer {TBST [1 M Tris–HCl, 0.15 M NaCl and 0.05% (v/v) Tween 20, pH 7.4]–3% (w/v) BSA}, the membranes were treated with mouse anti-HA/FLAG antibody (1:1000), or rabbit anti-GFP antibody (1:500) for 2 h. The membranes were washed four times (10 min each time) with TBST. After washing, the membranes were incubated with peroxidase-conjugated anti-mouse/rabbit antibody (1:2000) for another 2 h. The membranes were washed again and then detected with SuperSignal® West Dura substrate in a ChemiDoc™ MP Imaging System (Bio-Rad Laboratories, Inc., Richmond, CA, U.S.A.).

Glycosidase treatments were carried out according to the manufacturer's instructions with a few modifications. Protein samples were denatured at 50°C for 10 min and incubated with Endo H or PNGase F at 37°C for 2 h, followed by immunoblotting analysis as described above.

### Immunostaining and imaging

HEK293 cells were cultured on glass cover slips pre-coated with poly-D-lysine hydrobromide and transfected with the required plasmids. After 24 h of transfection, cells were fixed with 4% (w/v) paraformaldehyde for 15 min and washed. Then, they were incubated with blocking buffer containing 1% BSA for 1.5 h and treated with mouse anti-HA antibody (1:750) for another 1.5 h at room temperature. After washing three times with PBS, cells were treated with Alexa Fluor® 488-conjugated donkey anti-mouse antibody (1:400) for 1 h in the dark. After washing three times, cells were treated with Hochest 33342 and washed before being mounted with ProLong® Gold anti-fade reagent. Slips were then incubated at room temperature in the dark for at least 12 h and examined by an FluoView™ FV1000 confocal microscope (Olympus Corporation) using a 60× oil-immersion lens (488: λ_exc_=488 nm, λ_em_=510 nm; Hochest: λ_exc_=405 nm, λ_em_=422 nm). Images were captured with a CCD camera (charge-coupled-device camera) (Olympus).

For imaging HEK293 cells transiently expressing GLP-1R-GFP and ΔSP-GLP-1R-GFP, transfected cells (8×10^5^) were grown on pre-coated glass cover slips in 35-mm diameter dishes for at least 12 h. Cells were washed with warm PBS and incubated with CellMask™ Deep Red plasma membrane stain (1 μg/ml) for 20 min at 37°C. After washing, cells were immediately monitored by the confocal microscope with a 100 × oil-immersion lens (GFP: λ_exc_=488 nm, λ_em_=520 nm; Deep Red: λ_exc_=635 nm, λ_em_=668 nm).

### Real-time PCR

Twenty-four hours after transfection, total RNAs from HEK293 cells cultured in 6-well plates were extracted using Trizol® reagent. Two microgram of total RNA from each extraction were used as templates and cDNAs were obtained with High Capacity Reverse Transcription Kit. The resulting cDNAs were used as templates for real-time PCR analysis with the following oligonucleotides as the forward and reverse primers, respectively: 5′-CTACGTGAGCATAGGCTGGG-3′ and 5′-ATGGGCAGCCGGATAATGAG-3′. Samples were amplified in a ViiA™ 7 Real-Time PCR System (Applied Biosystems) under the following conditions: denaturing step at 95°C for 15 s, annealing step at 55°C for 30 s, extension step at 72°C for 30 s for 40 cycles. A comparative CT method was used to determine the relative level of GLP-1R transcription. Results were normalized to the endogenous GAPDH (glycerinaldehyd-3-phosphatdehydrogenase) control and compared with a reference sample (cells expressing the construct wt-GLP-1R).

### Data analysis

All the data were analysed using the GraphPad Prism software (GraphPad, San Diego, CA, USA). Non-linear regression analyses were performed to generate dose–response curves and to calculate IC_50_ and EC_50_ values.

## RESULTS

### Mutant GLP-1R without the putative SP was expressed with normal functionality

According to the SP prediction program ‘SignalP 3.0’ [18] or ‘SignalP 4.0’ [19], the sequence representing the first 23 amino acids of the GLP-1R fits all the criteria as an N-terminal SP. The putative SP possesses characteristic features such as a polar and charged N-terminal (n) region, a central hydrophobic (h) region and a polar C-terminal (c) region (Figure 1A). This sequence of GLP-1R is considered to be an N-terminal cleavable SP and an absolute requirement for receptor synthesis [16].

**Figure 2:**
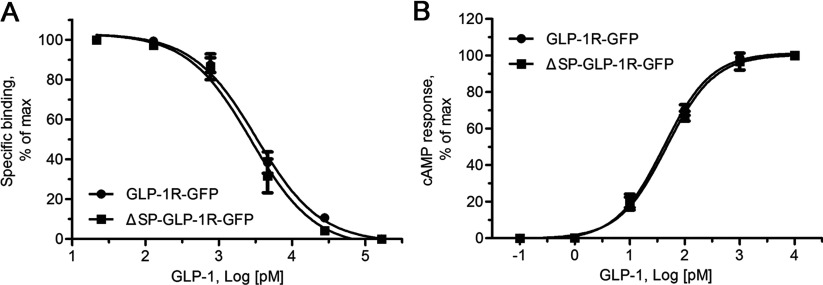
Pharmacological properties of the mutant GLP-1R lacking the putative SP with the wild-type receptor as a control (**A**) Binding curves for increasing concentrations of the unlabelled ligand GLP-1 (21.4 pM–166.7 nM) to displace binding of the radiolabelled ligand ^125^I-GLP-1 (200 pM) to HEK293 cells transfected with wild-type and mutant receptors. Values are calculated as percentages of maximal specific binding obtained in the presence of 21.4 pM unlabelled GLP-1. Non-specific binding and maximal specific binding of the mutant are similar to those of the wild-type (two-tailed paired *t* test). (**B**) Curves for intracellular cAMP responses in HEK293 cells to stimulation by increasing concentrations of GLP-1 (0.1 pM–10 nM). Values are calculated as percentages of maximal cAMP responses stimulated by 10 nM GLP-1. Basal and maximal cAMP levels of the mutant are similar to those of the wild-type (two-tailed paired *t* test). Data shown are means±S.E.M. from three independent experiments with each performed in triplicates.

To investigate the role of the putative SP, a ligand-binding study with mutant GLP-1R lacking the putative SP was performed to determine receptor expression. Plasmids encoding GLP-1R with or without the putative SP sequence were constructed (GLP-1R-GFP and ΔSP-GLP-1R-GFP; Figure 1B). GLP-1R-GFP with the putative SP was used as a control. Both receptors were C-terminally tagged with GFP to facilitate fluorescence and immunoblotting analyses. The constructs were transiently transfected in HEK293 cells. In whole-cell homogeneous competitive binding experiments, binding of ^125^I-GLP-1_7–36_ amide to the mutant GLP-1R was detected (Figure 2A). The affinity of the mutant GLP-1R was comparable with that of wild-type (IC_50_=3.43±0.77 and 3.01±0.95 nM, respectively; Table 1).

**Table 1:** Binding properties and bioactivities of wild-type and mutant GLP-1Rs Values are means±S.E.M., *n*=3. EC_50_ values were derived from cAMP measurements in HEK293 cells transfected with various GLP-1R constructs and stimulated by GLP-1_7–36_ amide. Binding affinities (IC_50_) were estimated using whole-cell preparations and with GLP-1_7–36_ amide as a control. Two-tailed paired *t* tests were performed for EC_50_ and IC_50_ values. No significant differences were found between the wild-type receptors (GLP-1R, GLP-1R-GFP and GLP-1R-HA) and the corresponding mutants (ΔSP-GLP-1R, ΔSP-GLP-1R-GFP and ΔSP-GLP-1R-HA).

Construct	Receptor binding (IC_50_, nM)	cAMP response (EC_50_, pM)
GLP-1R	1.60±0.46	101±17
ΔSP-GLP-1R	1.99±0.51	58±15
GLP-1R-GFP	3.43±0.77	41±8
ΔSP-GLP-1R-GFP	3.01±0.95	47±5
GLP-1R-HA	2.46±0.48	76±14
ΔSP-GLP-1R-HA	1.34±0.06	69±9

An adenylate cyclase activity study was carried out to examine the function of mutant GLP-1R without the putative SP. HEK293 cells transiently expressing ΔSP-GLP-1R-GFP could be stimulated by GLP-1_7–36_ amide and evoked cAMP production. Similar to the wild-type receptor, a typical cAMP accumulation curve was obtained for the mutant one (Figure 2B). EC_50_ values for the construct ΔSP-GLP-1R-GFP was comparable with that of GLP-1R-GFP (41±8 and 47±5 pM, respectively).

Experiments with C-terminally HA-tagged and untagged GLP-1Rs were additionally performed and similar results observed (Supplementary Figure S1; Table 1). These data indicate that mutant GLP-1R without the putative SP could be expressed in HEK293 cells with normal activities such as ligand (GLP-1) binding and eliciting downstream signalling event, namely, cAMP formation.

### Removal of the putative SP decreased the synthesis and glycosylation of GLP-1R

**Figure 3:**
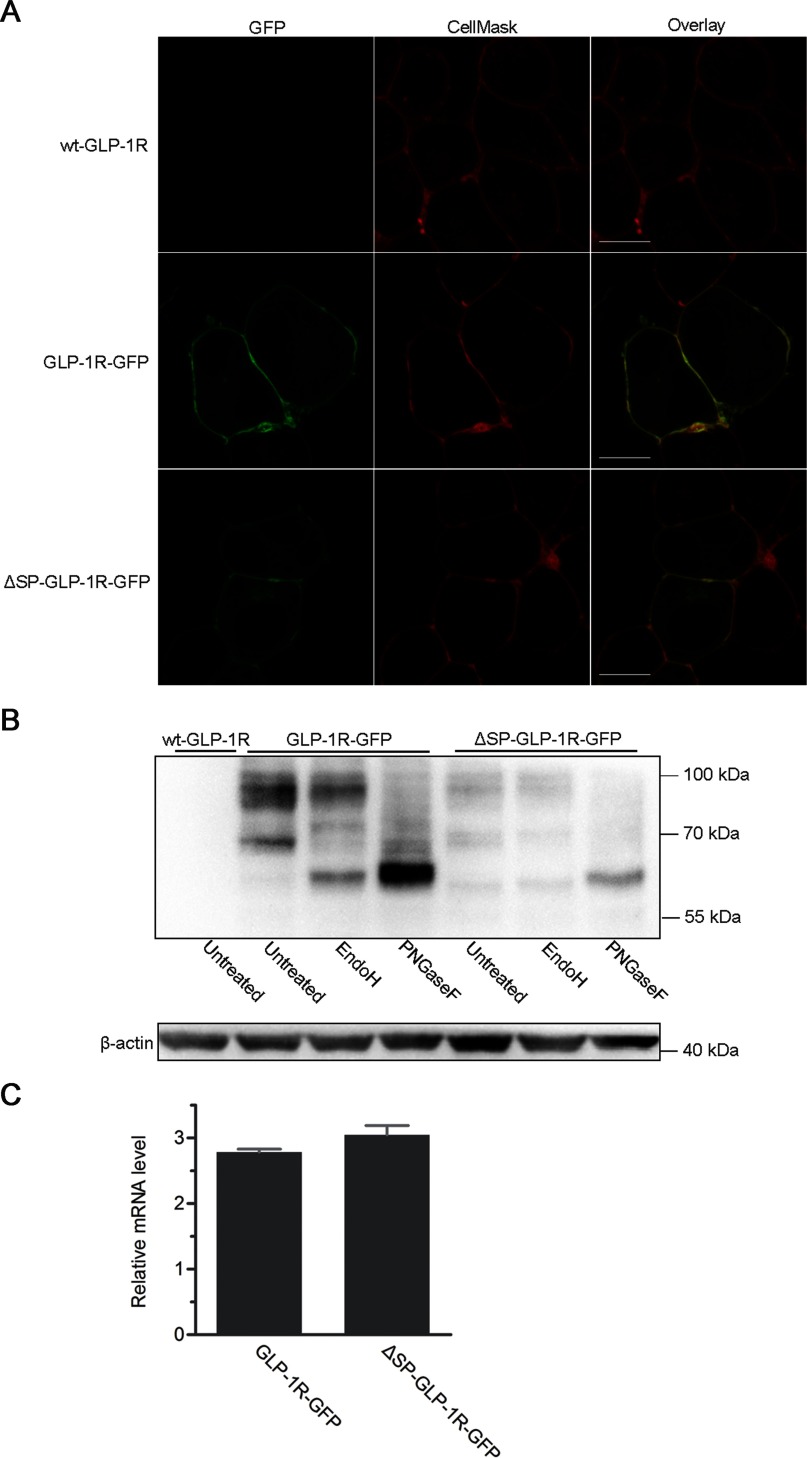
Comparison of GLP-1R-GFP and ΔSP-GLP-1R-GFP expression (**A**) Confocal laser scanning microscopy of HEK293 cells transiently expressing GLP-1R-GFP and ΔSP-GLP-1R-GFP with wild-type GLP-1R as a control. GFP intensity is shown in green (left panel) and membrane stained with CellMask™ Deep Red is displayed in red (central panel). Merge of both is exhibited in yellow (right panel). Horizontal scanning of representative cells is shown. Scale bar=10 μm. (**B**) Immunoblotting of GLP-1R expression and glycosylation in transiently transfected HEK293 cells. Lysates of cells expressing GLP-1R-GFP and ΔSP-GLP-1R-GFP were treated with Endo H or PNGase F to remove glycans or left untreated. Receptor proteins were detected with rabbit anti-GFP and HRP-linked goat anti-rabbit antibodies. Each lane shows a protein load from 5×10^5^ cells with β-actin as a control (detected with mouse anti-β-actin antibodies). The image is a representative of three independent experiments. (**C**) mRNA measurements were made in HEK293 cells transiently transfected with GLP-1R-GFP or ΔSP-GLP-1R-GFP. Relative mRNA levels were depicted as fold difference in mRNA expression after being normalized to the reference sample (cells expressing the wild-type GLP-1R) with GAPDH as an internal control. Data shown are means±S.E.M. from three independent experiments with each performed in triplicates. No statistically significant difference was found (two-tailed paired *t* test).

Expression of GFP-tagged receptors was monitored by confocal laser scanning microscopy and the fluorescence intensity detected in living, transiently transfected HEK293 cells. Membranes of the same cells were stained in red (Figure 3A, central panel), whereas receptor co-localization was shown in yellow (Figure 3A, right panel). For the wild-type GLP-1R, a strong GFP intensity was observed at the cell surface (Figure 3A, GLP-1R-GFP), while that of the mutant receptor without the putative SP was also visible, albeit it was weaker (Figure 3A, ΔSP-GLP-1R-GFP).

Wild-type and mutant GLP-1Rs were transiently expressed in HEK293 cells and their lysates collected for immunoblotting analysis. Anti-GFP and HRP-linked secondary antibodies were used to detect receptor proteins expressed in the cells. Two bands for the wild-type receptors were detected: a broad band with an apparent molecular mass of about 98 kDa plus a 69 kDa band (Figure 3B, GLP-1R-GFP). Diffusion of the broad band was probably caused by the complexity of the glycans linked to the receptor proteins. For the mutant receptors, these two bands were also detected with another visible band of 61 kDa (Figure 3B, ΔSP-GLP-1R-GFP). Untagged GLP-1R, used as a control, did not display these bands (Figure 3B, wt-GLP-1R). Immunoblotting with cell lysates collected at different time-points post transfection were performed and the results were similar (Supplementary Figure S2A).

Non-glycosylated and different forms of glycosylated GPCRs have often been detected in transfected cells [15,20]. GLP-1R contains three sites of N-linked glycosylation within the extracellular amino-terminal domain [12]. To better understand the immunoreactive protein bands, the glycosylation status of GLP-1R was determined. Receptors in cell lysates were treated with Endo H to remove high-mannose glycans, or with PNGase F to remove both complex and high-mannose glycans. The protein band of 98 kDa was resistant to Endo H treatment and its size was reduced to 63 kDa upon PNGase F treatment (Figure 3B, GLP-1R-GFP). The 69-kDa band was sensitive to the treatment of both Endo H and PNGase F, and was shifted to 63 kDa (Figure 3B, GLP-1R-GFP). This implies that the 98-kDa band represents the complex-glycosylated receptor; the 69-kDa band indicates the high mannose form of the receptor; and the molecular size of non-glycosylated receptor was about 63 kDa. In the case of the mutant receptor, situation was similar after Endo H or PNGase F treatment (Figure 3B, ΔSP-GLP-1R-GFP). However, the protein band of mutant GLP-1R was significantly weaker than that of the wild-type after PNGase F treatment (Figure 3B), suggesting that the total amount of mutant receptor proteins was reduced. In addition, non-glycosylated form of mutant GLP-1R was approximately 61 kDa in molecular size, a little smaller than that of the wild-type (Figure 3B).

To investigate the reduction of mutant GLP-1R synthesis in the HEK293 cells, its transcription level was assessed. Relative quantity of GLP-1R mRNA was determined by real-time PCR and the results did not reveal any significant difference between the mRNA levels of wild-type and mutant GLP-1R genes (Figure 3C).

### Epitope tags at the N-terminal of GLP-1R were detectable by immunofluorescence and immunoblotting

Signal peptide is usually cleaved after synthesis of a secreting or a membrane protein. As mentioned above, mutant GLP-1R was a little smaller in molecular size than wild-type receptor after PNGase F treatment. This indicates that wild-type GLP-1R protein may retain the putative SP. Cleavage of the SP could be demonstrated through detection of epitopes tagged at the N-terminal of a GPCR [16,21,22].

**Figure 4:**
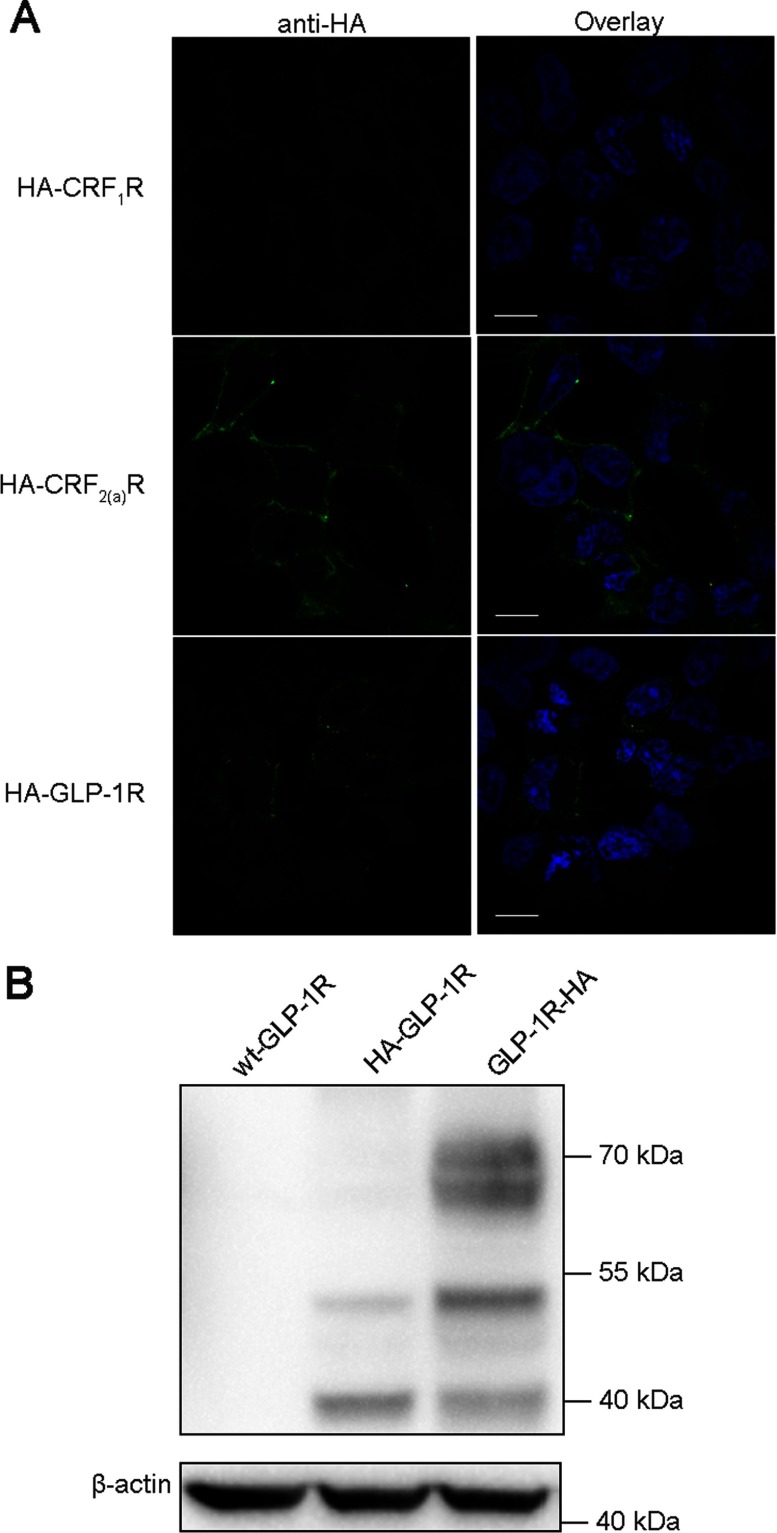
Detection of HA epitopes tagged at N/C-termini of different receptors expressed in HEK293 cells (**A**) Confocal laser scanning microscopy after immunostaining intact cells. HA-CRF_1_R, HA-CRF_2(a)_R and HA-GLP-1R constructs were transiently transfected and receptors at the cell surfaces were labelled with mouse anti-HA antibodies and Alexa Fluor® 488 donkey anti-mouse antibodies (left and right panels, green). Signal of Hochest 33342 staining is shown in blue (right panel). Horizontal scanning of representative cells is shown. Scale bar=10 μm. (**B**) Immunoblotting of lysates from HEK293 cells expressing wild-type GLP-1R, HA-GLP-1R and GLP-1R-HA. Receptor proteins were detected with mouse anti-HA and HRP-linked secondary anti-mouse antibodies. Each lane shows receptor proteins from 5×10^5^ cells with β-actin as a control. The immunoblotting shown is representative of three independent experiments.

GLP-1R, CRF_1_R and CRF_2(a)_R were N-terminally tagged with an HA epitope and transiently expressed in HEK293 cells. Anti-HA antibodies and Alexa Fluor® 488 secondary antibodies were used to detect the epitope of the receptors at the cell surface by indirect immunofluorescence with non-permeabilized cells. Green fluorescence intensity was detected for HA-CRF_2(a)_R but not for HA-CRF_1_R by confocal laser scanning microscopy (Figure 4A). This is consistent with the fact that the SP of CRF_1_R was cleaved after receptor synthesis and that of CRF_2(a)_R was not. Obvious green fluorescence intensity was detectable for N-terminally tagged GLP-1R (Figure 4A, HA-GLP-1R).

To confirm that the fluorescence intensity is indeed representative of GLP-1R proteins, immunoblotting analysis was performed to detect receptor proteins with anti-HA antibodies. The constructs, wt-GLP-1R, HA-GLP-1R and GLP-1R-HA, were transiently expressed in HEK293 cells and lysates of the cells were collected. The results demonstrated that there were three immunoreactive bands (approximately 40, 51 and 68 kDa, respectively; Figure 4B) for GLP-1R-HA (the weak 40-kDa band probably represented non-glycosylated receptors that were not visible for GLP-1R-GFP when detected with anti-GFP antibodies). For HA-GLP-1R, two protein bands were also detectable with the same molecular sizes of 40 and 51 kDa (Figure 4B). Results of immunoblotting with the cell lysates collected at different time-points after transfection were similar (Supplementary Figure S2B).

**Figure 5:**
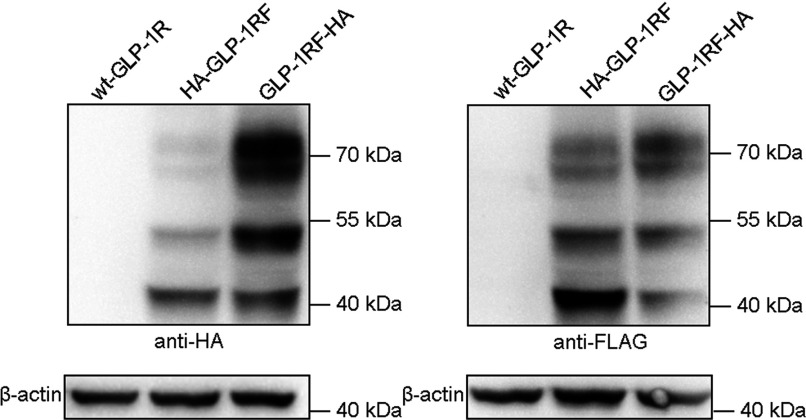
Immunoblotting analysis of the impact of epitope tagging on GLP-1R expression The indicated constructs were transiently expressed in HEK293 cells and the wild-type GLP-1R was used as a control. Receptor proteins were detected with mouse anti-HA antibodies (left panel) or mouse anti-FLAG antibodies (right panel) plus HRP-linked secondary anti-mouse antibodies. Samples for HA-GLP-1R-FLAG and GLP-1R-FLAG-HA are shown as HA-GLP-1RF and GLP-1RF-HA, respectively. Each lane shows receptor proteins from 5×10^5^ cells with β-actin as a control. Immunoblotting displayed is representative of three independent experiments.

The 68-kDa protein band of the N-terminally HA-tagged GLP-1R was scarcely detectable (Figure 4B, HA-GLP-1R). One of the explanations was that the N-terminal epitope influenced receptor expression and reduced complex-glycosylated receptors (68-kDa proteins). To unravel the impact of epitope tagging at receptor N-terminus, GLP-1R was first tagged with a FLAG epitope at the C-terminus to allow immunoblotting analysis and then tagged with an HA epitope at the N/C-terminus. The constructs, HA-GLP-1R-FLAG and GLP-1R-FLAG-HA, were transiently expressed in HEK293 cells and cell lysates were used for immunoblotting. Using anti-HA antibodies, patterns of protein bands for these two constructs were similar to those of HA-GLP-1R and GLP-1R-HA, and the upper band of HA-GLP-1R-FLAG was barely detectable (Figure 5, left panel). With anti-FLAG antibodies, the upper protein band for HA-GLP-1R-FLAG was much weaker than the other two bands, whereas that for GLP-1R-FLAG-HA was stronger than the other two bands (Figure 5, right panel). Patterns of protein bands with anti-FLAG antibodies remained similar, although the upper protein band for HA-GLP-1R-FLAG was detectable with anti-FLAG antibodies (Figure 5, right panel).

**Figure 6:**
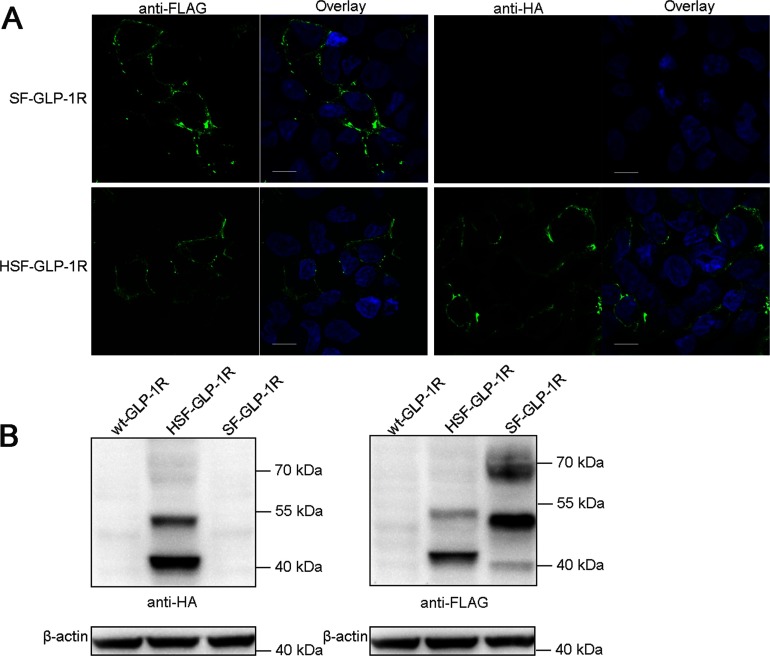
Immunofluorescence microscopy and immunoblotting analyses with the double-labelled GLP-1R (**A**) The constructs, HSF-GLP-1R and SF-GLP-1R, were transiently expressed in HEK293 cells and immunofluorescence microscopy was performed as described above. Signal of Alexa Fluor® 488 donkey anti-mouse antibodies is shown in green and that of Hochest 33342 staining in blue. Horizontal scanning of representative cells is shown. Scale bar=10 μm. (**B**) The indicated constructs were transiently expressed in HEK293 cells and wild-type GLP-1R was used as a control. Receptor proteins were detected with mouse anti-HA antibodies (left panel) or mouse anti-FLAG antibodies (right panel) by immunoblotting. Each lane shows receptor proteins from 5×10^5^ cells with β-actin as a control. Immunoblotting displayed is representative of three independent experiments.

To further investigate cleavage of the putative SP, GLP-1R was dual labelled with HA and FLAG epitopes at either sides of the SP. This construct was indicated as HSF-GLP-1R (Figure 1B). GLP-1R labelled with FLAG after the putative SP was indicated as SF-GLP-1R (Figure 1B). The constructs, SF-GLP-1R and wt-GLP-1R, were used as controls. HEK293 cells were transiently transfected with these constructs and immunofluorescence microscopy and immunoblotting analyses were performed. Epitopes on the receptors were detected using anti-HA/FLAG antibodies. Immunofluorescence microscopy with either anti-HA or anti-FLAG antibodies revealed that green fluorescence intensity at the cell surface was detectable for the construct HSF-GLP-1R (Figure 6A). Immunoblotting with either anti-HA or anti-FLAG antibodies revealed two detectable protein bands (approximately 42 and 53 kDa, respectively; Figure 6B) for this construct. However, for the construct SF-GLP-1R, only anti-FLAG antibodies could be used to detect the green fluorescence intensity or the protein bands (Figure 6). There were three protein bands for SF-GLP-1R with molecular sizes of approximately 40, 51 and 68 kDa, respectively. The two corresponding molecular sizes of HSF-GLP-1R were a little larger than those of SF-GLP-1R (Figure 6B), indicating that the receptors retained the putative SP.

## DISCUSSION

In the present paper, we were able to detect the binding of GLP-1_7–36_ amide to mutant GLP-1Rs without the putative SP in whole-cell binding assays when the mutant receptor was expressed in HEK293 cells. Activation of adenylate cyclase (cAMP accumulation assay) resulted from GLP-1_7–36_ amide stimulation in cells expressing the mutant receptor was also detected. Immunoblotting of the lysates from these cells (ΔSP-GLP-1R-GFP) revealed that the protein bands of this receptor were demonstrable (similar results were obtained with the construct ΔSP-GLP-1R-HA). Our data clearly exhibit that the SP of GLP-1R is not required for receptor synthesis and processing. It has been reported that most transmembrane domains of GPCRs can be used as a signal anchor sequence [13]. For the mutant GLP-1R without the putatvie SP, ER targeting is probably achieved by realization of the function of the first transmembrane helix (TM1) which acts as a signal anchor sequence. This phenomenon is also seen with other GPCRs such as the rat CRF_1_R and the human endothelin B receptor [4,14].

Until our work, the role of the putative SP of GLP-1R was considered to be an absolute requirement for receptor synthesis [16]. It was reported that proteins of mutant GLP-1R without the putative SP were not detectable by immunoblotting [16]. It was also claimed that there was no binding of GLP-1_7–36_ amide to membranes from HEK293 cells expressing the mutant receptor, nor coupling of the mutant receptor to activate adenylate cyclases [16]. However, in our experiments with a few modifications, the results were quite different.

Although the putative SP is not required for receptor synthesis, it still plays an important role in receptor synthesis and processing. Removal of the putative signal sequence of GLP-1R resulted in weaker protein bands in immunoblotting analysis, indicating a reduced receptor synthesis. Almost all of wild-type receptor proteins were glycosylated, while not all of mutant proteins were glycosylated because the protein band representing non-glycosylated proteins for the mutant receptor was detectable but that of the wild-type was not demonstrable (Figure 3B, ΔSP-GLP-1R-GFP). The counterpart in (1) CRF_1_R promotes receptor expression by improving one of the early steps of receptor biogenesis such as ER targeting and/or insertion; and (2) CRF_2(a)_R facilitates receptor trafficking through the early secreting pathway and consequently increases its cell surface presence [14,15]. It appears that the putative SP of GLP-1R exerts dual effects displayed by that of CRF_1_R and CRF_2(a)_R, respectively.

When the N-terminus of GLP-1R was tagged with an HA epitope and expressed in HEK293 cells, receptors at the cell surface could be lablled with anti-HA antibodies by immunofluorescence (Figure 4A). Immunoblotting also reveals two protein bands for the N-terminally tagged receptor although the pattern of the bands was different from that of GLP-1R-HA (Figure 4B). The reason for the difference is that epitope tagging influences the expression of this receptor and complex-glycosylated GLP-1Rs are largely reduced after epitope tagging at the N-terminus (Figure 5, right panel). Moreover, antigens near the functionally important extracellular N-terminal domain of GLP-1R, which has a folded structure, may be hard to detect. The two reasons above may explain why the upper band for HA-GLP-1R was not detected with anti-HA antibodies. The remaining two protein bands (40 and 51 kDa, respectively; Figure 4B, central lane) for the construct HA-GLP-1R were not detected in the previous work [16]. The putative SP of GLP-1R was then considered to be an N-terminal cleavable SP. But our results suggest that the putative SP might not be cleaved after receptor synthesis.

Consistent with the above is that non-glycosylated proteins of GLP-1R-GFP were a little larger in molecular size than ΔSP-GLP-1R-GFP proteins after PNGase F treatment (Figure 3B). The difference in molecular sizes is probably caused by retention of the putative SP which is about 2 kDa (23 amino acids) in wild-type receptors. Furthermore, the molecular weights between HA-GLP-1R-FLAG and GLP-1R-FLAG-HA were quite the same (Figure 5), whereas they were somewhat different between HSF-GLP-1R and SF-GLP-1R (Figure 6B), which also suggested the retention of the putative SP. If the putative SP was to be cleaved, the molecular weight of GLP-1R-GFP would be the same as that of ΔSP-GLP-1R-GFP, the molecular weight of HA-GLP-1R-FLAG would be different from that of GLP-1R-FLAG-HA, and the molecular weight of HSF-GLP-1R would be the same as that of SF-GLP-1R.

It is worth mentioning that both Huang and Bavec agreed that GLP-1Rs with an N-terminal GFP could be synthesized with the putative SP uncleaved [16,23]. Although the work by Huang showed that neither binding nor function was observed when GLP-1Rs with an N-terminal GFP and a C-terminal HA were expressed in HEK293 cells, the study by Bavec demonstrated that N-terminally GFP-tagged GLP-1Rs and N-terminally CFP (cyan fluorescent protein)/YFP (yellow fluorescent protein)-tagged GLP-1Rs could be translocated to the plasma membranes of CHO (Chinese-hamster ovary) cells with normal functionality [16,23,24]. The latter is in agreement with the fact that the putative SP of GLP-1R is not cleaved after receptor synthesis.

The maintenance of a putative SP is also seen with rat CRF_2(a)_R and human α_2C_-adrenoceptor, both possess an uncleaved putative SP [15,25]. However, the exact molecular mechanism underlying this phenomenon remains elusive. The cleavage site of an SP is characterized by small uncharged residues at positions -1 and -3 [6]. In the case of GLP-1R, there is a proline at position -1 from the predicted peptidase cleavage site, which might prevent the signal sequence from being cleaved [26,27]. When proline was replaced by alanine or serine, cleavage of the signal sequence did not occur, as the molecular sizes of the mutant receptors did not change and epitopes tagged at N-terminal of the mutants were detectable by immunoblotting (Supplementary Figure S3).

In conclusion, our results demonstrate that the putative SP of GLP-1R is not essential for receptor synthesis. The role of which was shown to promote both synthesis and processing of GLP-1R. This challenges our previous understanding about the action of this peptide. In addition, the present study also suggests that the putative SP of GLP-1R is probably not cleaved after receptor synthesis. Although cognate ligand binding and cAMP production were not affected by removal of this sequence, it may not be applicable to other ligands, particularly when an allosteric receptor modulator is present [28,29]. Moreover, according to the previous report, the uncleavable CRF_2(a)_R signal sequence points to an important role in downstream signal transduction [30]. Therefore further exploration of the putative SP on signalling pathways might yield new insights into GLP-1R biology.

## Online data

Supplementary data
