# Supplementary material for: The putative signal peptide of glucagon-like peptide-1 receptor is not required for receptor synthesis but promotes receptor expression
Source: Biosci Rep. 2014 Nov 21;34(6):e00152. doi: 10.1042/BSR20140120 (PMC4240022; doi:10.1042/BSR20140120)
Supplement: Supplementary data [file bsr034e152ntsadd.pdf]

Figure S1. Pharmacological properties of HA-tagged and untagged GLP-1Rs with or without the putative signal peptide. (A) and (C) Binding curves for increasing concentrations of the unlabelled ligand GLP-1 (21.4 pM-166.7 nM) to displace binding of the radiolabelled ligand ^{125}I-GLP-1 (200 pM) to HEK293 cells transfected with wild-type and mutant receptors. Values are calculated as percentages of maximal specific binding obtained in the presence of 21.4 pM unlabelled GLP-1. Non-specific binding and maximal specific binding of the mutant are similar to those of the wild-type (two tailed paired *t*-test). (B) and (D) Curves for intracellular cAMP responses in HEK293 cells transfected with wild-type and mutant receptors to stimulation by increasing concentrations of GLP-1 (0.1 pM-10 nM). Values are calculated as percentages of maximal cAMP responses stimulated by 10 nM GLP-1. Basal and maximal cAMP levels of the mutant are similar to those of the wild-type (two tailed paired *t*-test). Data shown are means ± S.E.M. from three independent experiments with each performed in triplicates.

Figure S2. Immunoblotting analysis of the cell lysates collected at different time-points post transfection. The constructs, wild-type GLP-1R, GLP-1R-GFP and ΔSP-GLP-1R-GFP (A), and wild-type GLP-1R, HA-GLP-1R and GLP-1R-HA (B), were transiently expressed in HEK293 cells. Cell lysates were collected at 24 h, 36 h and 48 h after transfection. Immunoblotting of the lysates was performed as described above. The image is representative of three independent experiments.

Figure S3. Impact of replacing the proline residue on the cleavage of the putative signal peptide. The proline residue of GLP-1R-GFP and HA-GLP-1R was replaced by alanine or serine and the corresponding receptors were transiently expressed in HEK293 cells. Immunoblotting analyses of receptor proteins were performed as described above. (A) The mutant receptors were indicated as P23A-HA-GLP-1R and P23S-HA-GLP-1R. wild-type GLP-1R, HA-GLP-1R and GLP-1R-HA were used as controls. Epitopes at the N-termini of the receptors were detected to assess cleavage of the putative signal peptide. (B) The mutant receptors were indicated as P23A-GLP-1R-GFP and P23S-GLP-1R-GFP. Wild-type GLP-1R, GLP-1R-GFP and ΔSP-GLP-1R-GFP were used as controls. Molecular sizes of non-glycosylated receptors (indicated by the arrow) were compared to assess cleavage of the putative signal peptide. Immunoblotting displayed is representative of three independent experiments.

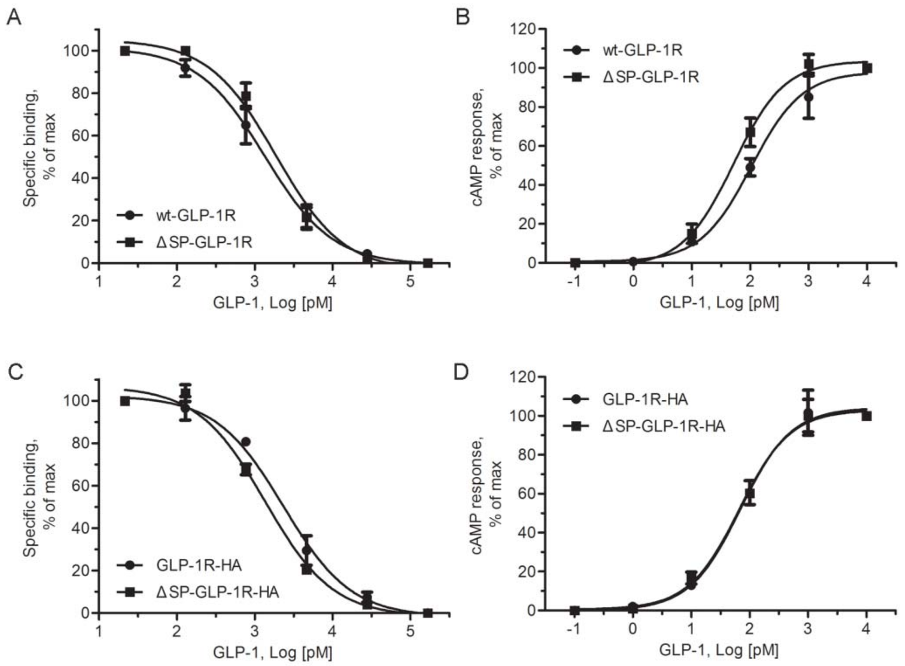

A
Specific binding, % of max
wt-GLP-1R
ΔSP-GLP-1R
GLP-1, Log [pM]
B
cAMP response, % of max
wt-GLP-1R
ΔSP-GLP-1R
GLP-1, Log [pM]
C
Specific binding, % of max
GLP-1R-HA
ΔSP-GLP-1R-HA
GLP-1, Log [pM]
D
cAMP response, % of max
GLP-1R-HA
ΔSP-GLP-1R-HA
GLP-1, Log [pM]

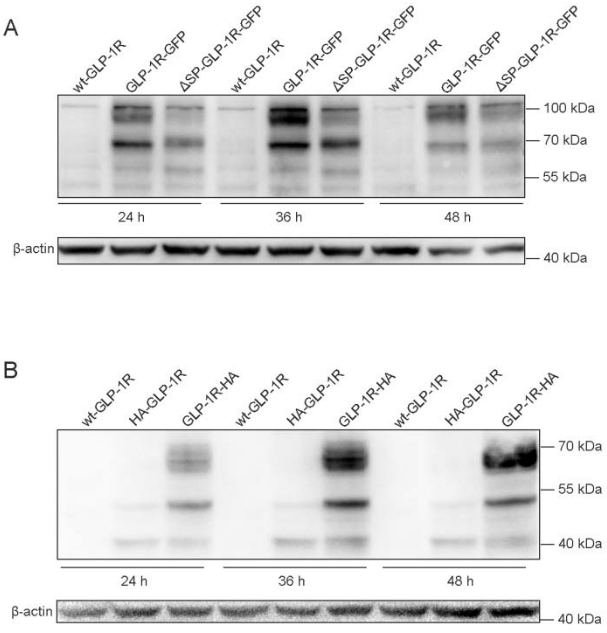
A
wt-GLP-1R
GLP-1R-GFP
ΔSP-GLP-1R-GFP
100 kDa
70 kDa
55 kDa
24 h
36 h
48 h
β-actin
40 kDa
B
wt-GLP-1R
HA-GLP-1R
GLP-1R-HA
70 kDa
55 kDa
40 kDa
24 h
36 h
48 h
β-actin
40 kDa

A

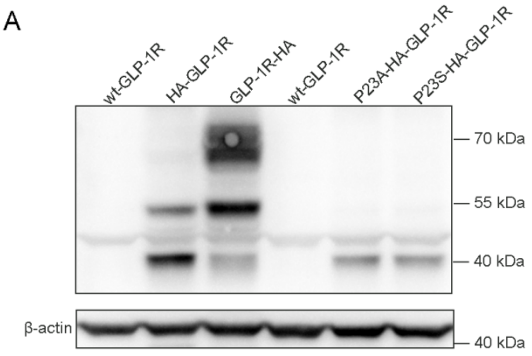

wt-GLP-1R
HA-GLP-1R
GLP-1R-HA
wt-GLP-1R
P23A-HA-GLP-1R
P23S-HA-GLP-1R
70 kDa
55 kDa
40 kDa
β-actin
40 kDa

B

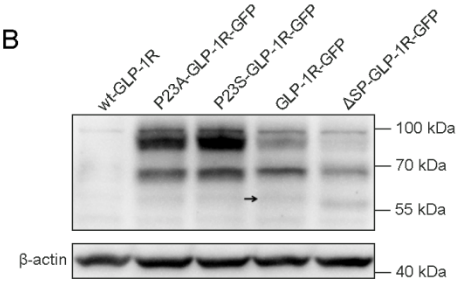

wt-GLP-1R
P23A-GLP-1R-GFP
P23S-GLP-1R-GFP
GLP-1R-GFP
ΔSP-GLP-1R-GFP
100 kDa
70 kDa
55 kDa
β-actin
40 kDa
